# Factors That Influence Prescribing in Borderline Personality Disorder: A Systematic Review

**DOI:** 10.1002/pmh.70014

**Published:** 2025-02-26

**Authors:** Joshua Confue, Ian Maidment, Sarah Jones, Matthew Jones

**Affiliations:** ^1^ University of Bath Bath UK; ^2^ Lincolnshire Partnership Foundation Trust Lincoln UK; ^3^ Aston University Birmingham UK

**Keywords:** borderline personality disorder, emotionally unstable personality disorder, prescribing, systematic review

## Abstract

Borderline personality disorder (BPD) is a psychiatric condition characterised by pervasive patterns of thinking and feeling, which can lead to social dysfunction and poor mental health. BPD has a significant impact not just on individuals with the diagnosis but also on those around them. Currently, no medication is licenced for BPD. Despite this, it is common for people with BPD to be prescribed multiple psychotropics. All psychotropic medications are associated with adverse events. A systematic review was conducted to explore factors that influence prescribing in adult BPD patients. Searches were conducted of EMBASE, PsycINFO, PubMed, EThOS and Web of Science. One‐hundred and two unique studies were identified, of which 13 suitable studies with diverse methodologies were included in the final synthesis. Of these, seven studies produced quantitative results, whereas the remaining six produced qualitative results. The synthesis identified several demographic factors statistically associated with prescribing. Most notably, prescribing was more likely in older patients and those with comorbid conditions. In addition to demographic factors identified, two key themes were generated from analysis of qualitative data from both healthcare professionals (HCPs) and patients discussing drivers: that the patient‐HCP relationship and the care pathway are crucial to the prescribing process from both perspectives. Prescribing medications for BPD is common, but there is limited data on the factors that affect this prescribing choice. HCPs must be aware of their own roles and perceptions in their relationships with BPD patients so that patients receive the most suitable treatment.

## 1 Introduction

Borderline personality disorder (BPD) is a psychiatric condition that falls under the category of personality disorders (World Health Organization 2019). Although we understand that labelling these complex presentations is controversial, we have used the term *borderline personality disorder* for clarity, because it is widely accepted and used in National Institute of Health and Care Excellent (NICE) Clinical Guidelines. It is characterised by pervasive patterns of thinking and feeling, which can lead to social dysfunction and poor mental health (Paton et al. 2015). Estimates of the prevalence of BPD in the general population vary, not only based on different diagnostic criteria and assessment processes but also across geographical locations (Shin et al. 2023). For instance, a 2006 review suggested that the weighted prevalence of the condition is 4.4% in the United Kingdom (Coid et al. 2006), whereas a 2014 study, based on data from the United States National Epidemiologic Survey on Alcohol and Related Conditions, estimated the prevalence in the United States to be about half this, at 2.7% (ten Have et al. 2016). Overall, population‐based survey studies show a prevalence range between 0.7% and 5.9% (Shin et al. 2023).

The condition can result in a high burden for patients, family members and healthcare systems (Bohus et al. 2021). One prospective follow‐up study found that four times as many patients with BPD die of suicide compared with the general population, with all‐cause mortality found to be three times higher in BPD patients (Temes et al. 2019).

The economic impact of personality disorders is challenging to estimate, but the costs are significant and thought to be rising (Botham et al. 2024). In England, a 2008 King's Fund report estimated the annual service cost per patient for those in contact with primary care teams at £286 (McCrone and King's Fund 2008). The total estimated annual cost was £704 million in 2007, along with additional loss of employment costs amounting to £7.9 billion (McCrone and King's Fund 2008). These figures were projected to rise significantly to £1.1 billion by 2026, with an alarming loss of employment cost of £12.3 billion (McCrone and King's Fund 2008).

A corresponding study in the Netherlands (with a population of about 15% of the UK's) estimated the societal costs of BPD to be around €2222 million yearly, only 22% of which were healthcare related. Although this study consists of a relatively small number of participants (88), it does provide data over a substantial period (14 years) and provides further evidence of the societal costs of this condition (van Asselt et al. 2007).

The evidence base for the use of medication in managing BPD is conflicted. The validity of BPD as a diagnostic construct is still disputed, and this may help explain some of the challenges in generating robust evidence in regards to its treatment (Tedesco et al. 2024). Currently, no medication is licenced for BPD in the United Kingdom, Europe or America (Abel et al. 2018; Silk 2015). It is also noteworthy that the American Association of Psychiatrists, the National Health and Medical Research Council of Australia and NICE of England, among other major institutions, concur on the recommendation of psychotherapy as the primary treatment for BPD, over psychotropic medication, due to the lack of sufficient evidence on the clinical effectiveness of medication (Bridler et al. 2015).

In particular, NICE's recommendation that psychotropic medication should not be used as a treatment for BPD draws upon the findings of an extensive, well‐structured 2010 Cochrane review (Lieb et al. 2010), which examined 27 trials to evaluate the effectiveness of pharmacological therapy in BPD. It concluded that mood stabilisers and second‐generation antipsychotics had the most beneficial effects. However, the authors noted that this conclusion was based mostly on single, small studies and that treatment should be symptom specific.

A 2022 update to this review included a further 18 trials. However, it once again found very low‐certainty evidence and concluded that medication most likely results in no difference in any primary outcome (Stoffers‐Winterling et al. 2022). Overall, the update supported the continued stance that no pharmacological therapy seems effective in treating BPD specifically (Stoffers‐Winterling et al. 2022).

In practice, however, it is common for people with BPD to be prescribed psychotropic medications. In 2014, the Prescribing Observatory for Mental Health (POMH‐UK) conducted a national audit in England, with all National Health Service organisations that provide specialist mental health services invited to report on prescribing in BPD. Of the 60 applicable organisations, 41 submitted information on 2600 randomly selected patients. The audit showed that over 70% of patients were prescribed an antipsychotic and more than 50% were prescribed a sedative, with these prescriptions overlapping in multiple cases (POMH‐UK 2014).

A 2015 inpatient study across Europe supports this picture of high prescribing levels. Of 2195 inpatients identified with BPD between 2001 and 2011, 70% were found to be prescribed antipsychotics, antidepressants or both, and 30% were prescribed benzodiazepines (Bridler et al. 2015). Perhaps most notably, over half of the patients received three or more psychotropic medications concomitantly (Bridler et al. 2015).

There is a noticeable contrast between the national guidance based on the current evidence base and the observed real‐world practice. To understand this discrepancy, it is essential to explore the factors that influence the prescribing of medication to patients with BPD. The existing evidence regarding the prescribing influences indicates a large number of potential factors (Schumock et al. 2004). It is worth noting at this juncture that there are a number of potential reasons why prescribing in psychiatric conditions may differ from other specialities. These reasons include, but are not limited to, diagnostic complexity, individualised treatment response and the subjective nature of psychiatric conditions. This review's aim was to evaluate the current practices and identify the factors that impact the prescribing of medication to BPD patients.

## 2 Methodology—Systematic

This review was conducted in line with the Preferred Reporting Items for Systematic Reviews and Meta‐Analyses (PRISMA) guidelines (Page et al. 2021) (compliance table Appendix A), and the protocol was registered on PROSPERO (Registration No. CRD42023422571). Ethical approval was not required, as the study only involved secondary analysis of anonymised data.

### 2.1 Research Question and Context

The research question was constructed through an iterative review of the topic of prescribing in BPD. The research team was comprised of four pharmacists with speciality, psychiatric and systematic review experience.

The use of structured, systematic search strategies helps to facilitate high‐quality research. As such, SPIDER was utilised, which was adapted from the PICO framework (Methley et al. 2014), given the expected results of mixed methods and qualitative research. The final research question is as follows:

What factors influence the decision to prescribe medication in the management of adult patients with borderline personality disorder?

### 2.2 Search Strategy

The review team developed a search strategy with input from a Health Sciences Librarian, which was subsequently peer‐reviewed by four supporting experts (Appendix B).

The following electronic information sources were searched: EMBASE (Elsevier), PsycINFO (EBSCOhost), PubMed (MEDLINE), Ethos (Grey Literature) and Web of Science. Searches were conducted on 11 September 2023 and rerun on 23 September 2024. Forward‐searching was conducted by manually reviewing the reference lists of included studies.

### 2.3 Inclusion and Exclusion Criteria

The criteria for study selection were generated based on mapping the research question, as described below.

The included studies

1. pertained to individuals who were 18 years and older and had been prescribed medication for, or with, a diagnosis of BPD, including individuals with comorbid psychiatric conditions (studies including patients aged < 18 years were included if relevant data could be extracted);
2. investigated pharmacological interventions, entailing all psychotropic medication utilised in the management of BPD, including but not limited to antidepressants, sedatives, benzodiazepines, antipsychotics and mood stabilisers (studies pertaining to herbal supplements and homoeopathy were not included);
3. explored or described factors influencing prescribing for medical and nonmedical prescribers. This included, but was not limited to, prescriber characteristics, patient characteristics, medication type and healthcare service design; and
4. were pieces of primary research of any design, published in full in English, conducted after the publication of the Diagnostic and Statistical Manual of Mental Disorders IV (1994), due to changes in diagnostic criteria between DSM‐III and DSM‐IV criteria (with the addition of the ninth criterion, ‘transient, stress‐related paranoid ideation or severe dissociative symptoms’) (Silk 2002).

### 2.4 Study Selection

After completing the searches, the data were imported into EndNote to remove duplicates. Subsequently, the remaining data were imported into Rayyan (www.rayyan.ai) for further processing. The study selection process consisted of two stages.

In Stage 1, all the studies' titles and abstracts were evaluated according to the inclusion and exclusion criteria. In case of doubt, studies were included at this stage and progressed to the next stage.

**FIGURE 1:**
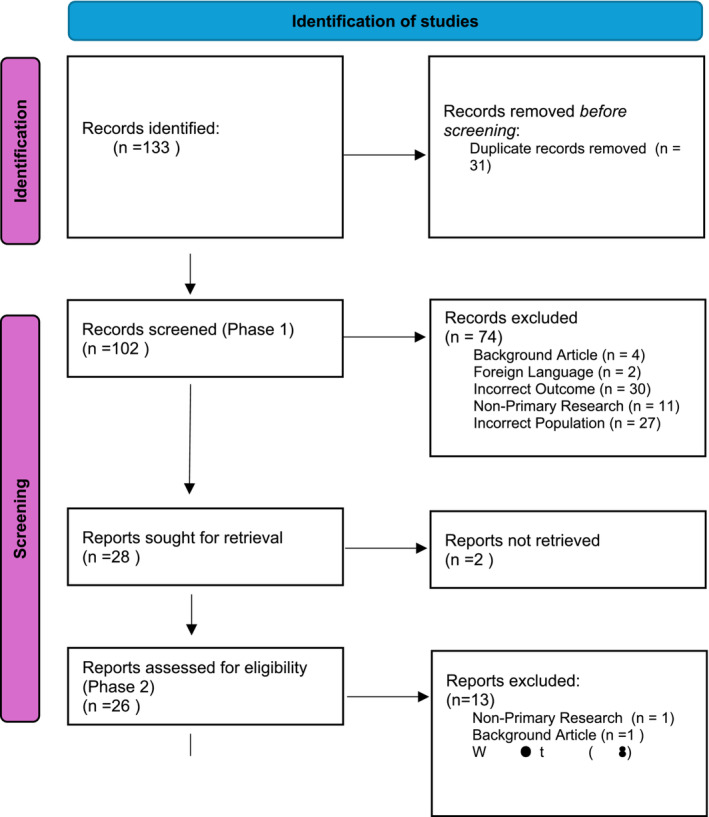
PRISMA diagram.

In Stage 2, the full texts of the retained studies were obtained and evaluated according to the inclusion and exclusion criteria. If the full text could not be obtained, the study was excluded. Any excluded study was documented, along with the reason for exclusion (Figure 1).

Two review team members (JC and SJ) made independent decisions regarding each study for both stages. If there was disagreement, they discussed and tried to reach a consensus. If a consensus could not be reached, a third member was consulted to make the final decision.

### 2.5 Data Extraction

An electronic data extraction form was created on a standardised Microsoft Excel spreadsheet based on the review question and objectives in consultation with all team members (Appendix C), following the guidelines provided by PRISMA (Page et al. 2021). Data were extracted by a single reviewer (JC) and then reviewed by a second reviewer (SJ).

### 2.6 Assessment of Quality

To assess the quality of each study selected for inclusion, the lead reviewer (JC) used the Mixed Methods Appraisal Tool (MMAT) (Nha Hong et al. 2018) to evaluate the risk of bias in each study. Studies were grouped based on qualitative or quantitative results before being assessed based on their described methodology.

### 2.7 Analysis

Following the completion of searches, it was determined that meta‐analysis was an unsuitable approach due to the significant heterogeneity in the studies selected for inclusion. Therefore, the team summarised the findings through a narrative synthesis, focused on the prescribing processes. This methodology allowed for a flexible approach, encompassing the range of methodologies, populations, and results reported in the identified studies (Ryan 2016; Sukhera 2022).

Findings from the included studies were analysed through results‐based, convergent synthesis design (Noyes et al. 2019). Quantitative studies with numerical results were analysed using numerical analysis; descriptive statistics were utilised, and the results were grouped according to prescribing factors. Thematic data were analysed using a modified inductive thematic analysis approach based on Braun and Clarke's method (Byrne 2022). Qualitative outcome studies were reviewed to derive codes and subcodes representing information useful in addressing the research question by the lead reviewer (JC). These codes were then developed into descriptive themes and grouped by the stakeholders' nature (healthcare professional [HCP] or patient/carer) with input from four members of the team (JC, SJ, IM and MJ). Subsequently, these themes were defined and interpreted to develop a descriptive format.

Finally, the project team combined the results of both numerical and thematic analyses into the narrative discussion. The ENTREQ reporting guidelines were used for this review (Tong et al. 2012) (Appendix D).

## 3 Results

The searches identified 133 records, from which 13 studies were selected for inclusion in the review (Figure 1). Table 1 presents an overview of the included studies. The selected articles demonstrated significant variations in study design, including semistructured interviews, Delphi studies, quantitative cross‐sectional surveys and retrospective observational studies. The included studies were predominantly conducted in the United Kingdom, with additional studies from Europe, Iran and the United States. The number of participants varied widely, with sample sizes ranging from 9 to 550 participants (Table 1).

**TABLE 1:** Included studies characteristics.

	Pascual et al. (2007)	Pascual et al. (2010)	Crawford et al. (2011)	Knappich et al. (2014)	Tong et al. (2021)	Pascual et al. (2021)	Mirhaj Mohammadabadi et al. (2022)	Martean and Evans (2014)	Dickens et al. (2016)	Wlodarczyk et al. (2018)	Patel and Konstantinidou (2020)	Schulkens et al. (2021)	Javed et al. (2022)
Research outcome type	Numerical	Numerical	Numerical	Numerical	Numerical	Numerical	Numerical	Thematic	Thematic	Thematic	Thematic	Thematic	Thematic
Study design	Retrospective observational Study	Retrospective naturalistic study	Retrospective observational study	Questionnaire	Observational cross‐sectional study	Observational cross‐sectional study	Cross‐sectional questionnaire	Semistructured interview	Semistructured interview	Focus groups	Focus groups	Delphi	Questionnaire
Country/countries from which study participants were recruited	Spain	Spain	United Kingdom (England)	Germany	United Kingdom	Spain	Iran	United Kingdom (England)	United Kingdom (England)	South Australia	United Kingdom	Netherlands, Belgium, United Kingdom, United States, Switzerland, Australia	United Kingdom (England)
Setting	Secondary care psychiatric services (emergency)	Secondary care psychiatric services	Secondary care psychiatric services	Private psychiatric practice	Across services	Secondary care psychiatric services	Secondary care psychiatric services	Secondary care psychiatric services	Secondary care psychiatric services	Primary care services	Secondary Care psychiatric services	Across settings	Secondary care psychiatric services
Participants	Patients	Patients	Patients	Consultant Psychiatrists	Patients	Patients	Patients	Consultant psychiatrists	Patients	GPs, university‐based researchers, psychiatrists, PATIENTS	Patients (in group therapy)	Experts (inclusion criteria provided in study, but breakdown of participants not reported)	Medical prescribers (consultants, staff grade doctors or higher level trainees)
Number of participants	540	226	332	141	51	620	64	11	20	22 (12 GPs, 10 other stakeholders)	7 (4 and 3 across two focus groups)	18	9
Age	Mean age 31.3	Mean age 28.9	Mean age 42.5	Mean age 51.7	Mean age 35.7	Mean age 30.4	Mean age 33.6	Not provided	Mean age 27.8	Not provided	20–54 (Mean age not provided)	Not provided	Not provided
% Female	11.7%	85.8%	49.4%	59.0%	39.2%	88.0%	84.4%	Not provided	100.0%	Not provided	57.1%	Not provided	Not provided
% Male	88.3%	14.2%	50.6%	41.0%	60.8%	12.0%	15.6%	Not provided	0.0%	Not provided	42.9%	Not provided	Not provided

Abbreviation: GP, general practitioner.

Seven studies produced numerical results reporting on factors associated with prescribing, two of which also reported on the value of treatment adherence. Table 2 provides a summary of these results. Six studies produced thematic results reporting themes related to the prescribing process, as summarised in Table 3.

**TABLE 2:** Numerical outcomes.

	Pascual et al. (2007)	Pascual et al. (2010)	Crawford et al. (2011)	Knappich et al. (2014)	Tong et al. (2021)	Pascual et al. (2021)	Mirhaj Mohammadabadi et al. (2022)
	Factors associated with prescription	Factors associated with prescription	Factors associated with prescription	Value of pharmacotherapy	Factors associated with prescription	Factors associated with prescription	Treatment adherence to pharmacological intervention
Outcome details general Factors influencing prescribing regardless of medication class			> 80% of patients prescribed medication Presence of comorbid condition is the biggest predictor of prescribing. Medication prescription is more likely in patients with: ‐ Comorbid depression (OR 3.0, CI 95% 1.4–6.5) ‐ Substance misuse (OR 3.0, 95% CI 1.4–6.5). ‐ Specialist PACT Treatment (OR 0.35, CI 95% 0.13–0.95)		50% of patients were on three or more medications BPD patients are more likely to self‐harm (OR 2.3, *p* = 0.005) (x29.204, *p* = 0.002) BPD patients are not more likely to have forensic history (x2 = 0.337 *p* = 0.561) BPD patients are not more likely to suffer from alcohol or illicit substance abuse (statistical analysis not provided)	Medication prescription is more likely in: ‐ older patients (mean age 30.9 vs. 27.3 *p* = 0.0002) ‐ patients with comorbid psychiatric conditions: 77.8% vs. 58.2% (OR 2.5, 95% CI 1.5–4.2) Polypharmacy is more likely in: ‐ older patients (mean age 31.9 vs. 29 *p* < 0.0001) ‐ patients with comorbid psychiatric condition: 83.2% vs 67.5% (OR 2.4, 95% CI 1.6–3.6) The regression model associated the following factors with polypharmacy:‐ affective, anxiety, and eating disorder symptoms (AUC 0.707, 95% CI 0.667–0.748)	Gender, occupation, education, marriage, and hospitalisation have no effect on self‐reported adherence to medication
Factors influencing prescribing of antidepressants		Antidepressant prescription is associated with age, comorbid conditions, affective and anxiety symptoms Antidepressant prescription is nearly three times as likely in the presence of comorbid anxiety disorder (OR 2.77, 95% CI 1.16–6.1) SSRI prescriptions associated with eating disorders (OR 2.01, 95% CI 1.12–3.61)			Antidepressants were the most commonly prescribed agent > 90% of BPD patients were prescribed one or more antidepressants		
Factors influencing prescribing of benzodiazepine	Benzodiazepine prescription is more likely in: ‐ male patients (female OR 0.63, 95% CI 0.42–0.88) ‐ patients with anxiety (OR 3.77, 95% CI 2.52–5.66) Benzodiazepine prescription is less likely in patients with: ‐ issues with self‐care (OR 0.61, CI 96% 0.42–0.88) ‐ drug problems (OR 0.58, CI 95% 0.38–0.88)	Benzodiazepine prescription is more likely in: ‐ older female patients () Benzodiazepine prescription is less likely in patients with: ‐ substance misuse disorder comorbidity (OR 0.45, 95% CI 0.24–0.87)		Benzodiazepines were prescribed by 71.4% of psychiatrists 60% of respondents selected benzodiazepines for their anxiety‐reducing effects. 16% of respondents selected a benzodiazepine based on the shortness of its half‐life.			
Factors influencing prescribing of mood stabilisers		Mood stabiliser prescription is more likely in: ‐ older patients with less severe symptoms (based on DIB‐R scale) ‐ the presence of comorbid anxiety disorder (OR 2.95, 95% CI 1.04–3.69) ‐ patients with previous hospitalisation (OR 2.82, 95% CI 1.48–5.36)		Mood stabilisers were prescribed by 74.6% of psychiatrists Agent most commonly prescribed was valproate (no reason given)			
Factors influencing prescribing of antipsychotics	Antipsychotic prescription is more likely in: ‐ male patients (female OR 0.63, CI 95% 0.43–09.4) ‐ patients presenting higher risk to others (OR 2.07, CI 95% 1.39–3.06) ‐ patients with a history of drug use (statistics not provided) ‐ patients with psychosis in symptomatology (OR 6.88, 95% CI 1.64–28.90)	Antipsychotic prescription is more likely in: ‐ older men with higher impulsivity and affective scores, and substance misuse ‐ patients with previous hospitalisation (OR 1.89, 95% CI 1.0.1–3.54) ‐ patients with higher impulsivity scores (OR 1.77, 95% CI 1.09–2.87)		92.2% of respondents selected a second generation antipsychotic 70.1% of respondents stated that the agent most commonly prescribed was quetiapine (no reason given)			

Abbreviations: AUC, area under curve; BPD, borderline personality disorder; CI, confidence interval; DIB‐R, Diagnostic Interview for Borderlines (Revised); OR, odds ratio; PACT, Personality and Complex Trauma team.

**TABLE 3:** Thematic outcomes.

	Dickens et al. 2016	Martean and Evans 2014	Wlodarczyk et al. 2018	Patel and Konstantinidou 2020	Schulkens et al. 2021	Javed et al. 2022
	Themes	Themes (and subthemes)	Themes, challenges and strategies	Themes	Treatment algorithm	Themes
Number of Outcomes	4 themes	4 themes	4	5	4 algorithm nodes	13
Themes influencing prescribing in patients with BPD	Evaluation (P)	Difficulty collaborating in emotionally charged situations (H)	Challenges around diagnosis (H)	Medication has a powerful impact on the body and mind (P)	Presence of cognitive‐perceptual symptoms	Symptomatic treatment (H)
	Wellbeing (P)	Feeling helpless, unable to relieve suffering (H)	Comorbidities and clinical complexity (H)	Confronting the powerful position of doctors (P)	Impulsive behaviour	Balancing psychological and pharmacological treatment (H)
	Understanding (P)	Drugs as a facilitator of the doctor–patient relationship (H)	Difficulties with patient behaviour and GP relationship (H)	[Medication as a] moderator to the prescribing experience(P)	Affective dysregulation	Treating other comorbidity (H)
	Self‐management (P)	Effects of discontinuing [medication] on doctor‐patient relationship (H)	Finding and navigating the system for support (H)	[Patients'] expectations of healthcare professional (P)	Previous treatment	Feeling pressured [from] high expectations from service users (H)
				Needing a relationship with the doctor (P)		NICE guidelines restrict clinicians' prescribing (H)
						Limited evidence for prescribing
						Clinicians fear intensifying feelings of anger from not prescribing and self‐harm (H)
						Practical challenges [of] following guidelines (H)
						Evidence‐based prescribing (H)
						Experience based on service users' previous response (H)
						Feeling uncomfortable/discomfort due to patient response (H)
						Feeling stuck with established prescriptions (H)
						Inadvertent risk (H)

*Note:* Bracket words in themes have been added to provide context and clarity.

Abbreviations: GP, general practitioner; (H), health care professional; NICE, National Institute of Care and Clinical Excellence. (P), patient.

### 3.1 Risk of Bias and Quality Assessment

The MMAT focuses on five sets of criteria for different study designs. This enables the evaluation of methodological distinctions in studies within the review using a single tool (Nha Hong et al. 2018). Following evaluation, all 13 studies were included in the synthesis, with more weight given to more rigorous studies in the thematic analysis (Appendix E). No randomised controlled studies were found, and the variation in the assessment of results and the definition of medication groups added complexity to the interpretation. Most of the identified studies had relatively small sample sizes, and even the larger studies were often retrospective or observational, limiting the conclusions that could be drawn. The overall robustness of the findings must be considered low, largely due to the scarcity of available data.

### 3.2 Demographic Factors

The section below describes quantitative data, regarding factors associated with prescribing in BPD. Six patient demographics were associated with medication prescribing in BPD: comorbidity, age, hospitalisation, gender, presentation of risk and severity of presentation. Only comorbidity and age were reported to be associated with prescribing regardless of medication class.

#### 3.2.1 Comorbidity

Comorbidity—here defined as the presence of an additional psychiatric condition—was associated, in two identified studies, with an increased probability of prescribing regardless of medication class utilised. In both these studies, comorbid psychiatric conditions were identified as a significant predictor of prescribing (Crawford et al. 2011; Pascual et al. 2021).

A 2011 UK study found that comorbid depression was the most significant predictor of prescribing, with an odds ratio (OR) of 3.0 (95% confidence interval [CI] 1.4–6.5) (Crawford et al. 2011). This conclusion was further supported by a subsequent 2021 study of over 600 records, which found that individuals were more likely to be prescribed medication if they had a comorbid psychiatric condition (77.8% vs. 58.2%) (Pascual et al. 2021).

However, certain comorbid conditions were associated with the prescribing of specific medication classes. One study reported that antidepressant prescribing was more likely in the presence of comorbid conditions, particularly anxiety‐related disorders (OR 2.77, 95% CI 1.16–6.1) (Pascual et al. 2010). Comparatively, antipsychotic prescribing was found to be more common in the presence of psychosis symptomatology (OR 6.88, 95% CI 1.64–28.90) (Pascual et al. 2007). This study also found that benzodiazepine use was more common in those with anxiety (OR 3.77, 95% CI 2.52–5.66) (Pascual et al. 2007).

Notably, some factors were associated with reduced incidence of prescribing. A 2010 study found that patients prescribed benzodiazepines were less likely to have addiction issues than those who were not (OR 0.45, 95% CI 0.24–0.87) (Pascual et al. 2010). This is supported by an earlier study from 2007, which found that benzodiazepine use was more common in those with fewer drug problems (OR 0.58, CI 95% 0.38–0.88) (Pascual et al. 2007). This supports the idea that drug addiction problems may lower prescribing of benzodiazepines.

Mood stabilisers, which are an ill‐defined medication class with significant overlap with antipsychotics and anticonvulsants, were found in a 2010 study to be more commonly utilised in patients with comorbid anxiety disorders (OR 2.95, 95% CI 1.04–3.69) (Pascual et al. 2010). In this study, mood stabilisers were not defined by the author but were said to largely consist of topiramate (72% of these patients) and valproate (21%).

Overall, it appears that the presence of comorbid conditions, and perhaps anxiety in particular, is associated with increased levels of prescribing.

#### 3.2.2 Age

Age was the second variable associated with prescribing regardless of medication class, though this conclusion is based on limited data. A 2021 study found that individuals were more likely to be prescribed medication, regardless of class, if they were older, with a mean age of 30.9 versus 27.3 years (*p* = 0.0002) (Pascual et al. 2021).

In particular, mood stabilisers—which, as noted above, are a poorly defined group of medications—(Malhi et al. 2018) were also found to be more commonly prescribed to older individuals, though specific statistics were not provided (Pascual et al. 2010). Again, the authors did not define mood stabilisers, but they were said to largely consist of topiramate (72% of these patients) and valproate (21%).

#### 3.2.3 Hospitalisation

Although not associated with general increases in prescribing, a single 2010 study found an increased likelihood of antipsychotic prescribing (OR 1.89, 95% CI 1.01–3.54) in hospitalised patients (Pascual et al. 2010). Additionally, the same study found mood stabiliser prescribing was also more common in those with a history of previous hospitalisation (OR 2.82, 95% CI 1.48–5.36) (Pascual et al. 2010).

#### 3.2.4 Gender

Two studies found associations between gender and the prescribing of particular classes of medication.

Two separate studies conducted in Spain identified that antipsychotics were more frequently prescribed to male patients than to female patients (Pascual et al. 2007, 2010). The 2007 study identified the prescribing of antipsychotics as less likely in female patients (female OR 0.63, CI 95% 0.43–09.4); the subsequent 2010 study concurred with this conclusion but did not provide specific statistics to support its findings.

The evidence around benzodiazepine usage and gender, however, appears conflicted. The 2007 study found that benzodiazepine use was also more common among male patients than female patients (female OR 0.63, 95% CI 0.42–0.88) (Pascual et al. 2007). This conclusion is contradicted by the 2010 study, which suggested, from a multivariate analysis, that benzodiazepines were more common among older female patients than male patients, though specific statistics were again not provided (Pascual et al. 2010). This study, however, may have been biassed due to gender skew in the sample group, as 85.8% of the patients were female.

#### 3.2.5 Presentation of Risk

At best, ‘risk’ is ill‐defined as a term in clinical practice; however, two studies reported its correlation with prescribing. One study found antipsychotic prescribing more likely in patients assessed as presenting a risk to others (OR 2.07, 95% CI 1.39–3.06) (Knappich et al. 2014), whereas a second study found that benzodiazepine use was less common among patients presenting fewer self‐care issues (OR 0.61, CI 96% 0.42–0.88) (Pascual et al. 2007).

#### 3.2.6 Severity of Presentation

Only one study reported on associations between severity of presentation and prescribing. This 2010 study reported that higher impulsivity scores were associated with the use of antipsychotics (OR 1.77, 95% CI 1.09–2.87) (Pascual et al. 2010), whereas the use of mood stabilisers was more common in those with less severe symptoms based on the Revised Diagnostic Interview for Borderlines (DIB‐R) scale (Pascual et al. 2010). The DIB‐R scale (Zanarini et al. 1989) is a semistructured clinical interview used to diagnose BPD (Carcone et al. 2020).

### 3.3 Thematic Factors

In addition to factors identified through the numerical analysis, the review identified two key themes associated with the prescribing of medication to patients with BPD: relationships and the care pathway.

#### 3.3.1 Relationships

Generally, across the identified literature, relationships were identified as an integral part of the prescribing of medication by both HCPs and patients.

In many countries, HCPs are taught that it is important to discuss treatment options carefully with patients and to ensure that patients are involved in the prescribing process (Say and Thomson 2003). It is, therefore, logical that the patient would emerge as a central factor in the prescribing process. Nevertheless, it is important to note that many of the reported themes from the reviewed studies appear to imply negative connotations around this relationship ‐ denoted by the terms ‘difficulty’ and ‘challenging’. ‘Difficulty collaborating with patients’ was identified as a significant factor in the prescribing process by three of the four studies exploring the views of HCPs (Martean and Evans 2014; Wlodarczyk et al. 2018; Javed et al. 2022) with comments such as

troubled individuals. (Wlodarczyk et al. 2018)

Sometimes they will be really angry or upset if they are not getting what they want. (Martean and Evans 2014)

It just felt to me that the projection was so strong. (Martean and Evans 2014)

These quotations suggest that some HCPs perceive patients with a diagnosis of BPD as challenging to engage with, particularly in the context of shared‐decision making.

The number of studies exploring patients' perspectives was limited to two studies (27 participants in total). Although this represents a limited data pool, the analysis showed that the HCP–patient relationship is also a significant theme for patients. The theme of ‘relationships’ differed for patients, focusing more on the ‘need’ for a strong relationship and less on the relational challenges. Strong relationships were described using terms related to understanding, continuity of care, ease and degree of access to the HCP. The included studies identified subthemes of ‘needing a relationship’ and the ‘desire to confront authority’.

Back in the day, that sounds really silly, but you used to have one GP …

I think you should be able to book an appointment with your doctor [GP] that lasts 15–20 min. (Patel and Konstantinidou 2020)

The idea of ‘need’ is prominent in both of the identified studies relating to patients' perspectives on BPD treatment. This ‘need’ could be understood as patients' desire to be prescribed medication, referred to specialist service, or otherwise validated. It may also be part of the reason for the different focuses in referring to the HCP–patient relationship for each party; patients need or seek something from HCPs, and therefore, this is a singularly important relationship for patients. For HCPs, however, the HCP–patient relationship is one of many equally important relationships with other patients. This may be the cause of the perceived ‘difficulty’ of treating BPD patients, indicated by comments such as

Sometimes I've got a patient that I think is a bottomless pool of need. (Wlodarczyk et al. 2018)

People are people, they're not diagnoses. So, you'll keep on seeing them … and you know that, yep, she's going to be needy and you hope that one day she'll say, ‘yeah, today's a good day’. (Wlodarczyk et al. 2018)

I've made an hour‐long appointment for a patient because she has so many problems, but then she didn't turn up. (Wlodarczyk et al. 2018)

#### 3.3.2 Care Pathway

HCPs do not act in a vacuum. In modern healthcare, they are one of a number of professionals engaging with patients. ‘Integrated care’ or ‘disease‐management’ pathways detail the fundamental steps in patient care and outline patients' expected journey through the healthcare system (Campbell et al. 1998). They are long processes that involve initial contact, follow‐up, referral to and from specialists and often a return to primary care (Schrijvers 2009). It should, therefore, not come as a surprise that both HCPs and patients felt these pathways influenced patients' care and the prescribing of medication.

The review identified a recurrent theme of ‘helplessness’ on the part of HCPs for a multitude of reasons, from lack of access of services, difficulties implementing national guidelines and feeling unable to provide care for patients with BPD.

What can you offer to them? (Martean and Evans 2014)

We must have a consensus statement on what the evidence‐based interventions are that you can use in these conditions. Most diseases have a manual plan …. (Wlodarczyk et al. 2018)

The implication here is that, fundamentally, HCPs are unsure or unable to address the needs of the patient despite the presence of guidelines. Though it is not possible to determine from secondary analysis, it is worth considering how this ‘helplessness’ plays into the HCP–patient relationship.

Another key theme that emerged from the studies was that of ‘uncertainty’ on the part of HCPs, particularly around the management of BPD. HCPs may find it difficult to manage ‘uncertainty’, as they might fear their patients' reactions and wish to avoid losing the trust of the patient; this may lead them to avoid expressing their uncertainty openly (van der Bles et al. 2019). Additionally, if they are ‘uncertain’ of the care pathway, they may find it difficult to manage the patient's expectations.

Investigation into the perspective of patients with BPD found that, when attending appointments, patients often had specific expectations regarding their care and the healthcare system, such as being referred to specialist services or being prescribed specific medications (Patel and Konstantinidou 2020) (Dickens et al. 2016). It is unclear why some patients have focussed expectations regarding pharmacological treatment, although some patient comments show clear expectations of benefits:

The reason why I struggled as much as I did was that my GP never did want to put me on anything. (Patel and Konstantinidou 2020)

When you take the medications that are prescribed to you, it gives you energy, it clears your mind, it allows you to tolerate difficult feelings, distressing feelings, and it allows you to engage in some of the work that you do here, well, by being on medication. (Patel and Konstantinidou 2020)

Medication … gets you to a point where you can be reached by other people. (Patel and Konstantinidou 2020)

Moreover, a 2020 study into experiences in treating BPD reported that patients might become ‘angry and exhibit self‐destructive behaviours when they feel neglected by their doctor’ (Patel and Konstantinidou 2020). This indicates the importance of understanding patient expectations in any HCP‐patient relationship, highlighting a crossover between the two key themes identified across the studies.

I went in and asked to talk to my psychiatrist that was supposed to be there and they were like ‘He's not here today’ […] basically you are telling us that we are [s]upposed to pick and choose when we are gonna have a really bad day or really bad thoughts. (Patel and Konstantinidou 2020)

Patients want to feel they can rely on or trust their care team, linking back to a relationship between the HCP and the patient.

## 4 Discussion

Analysis of the included literature identified several demographic factors statistically associated with prescribing in BPD, such as comorbid conditions, older age and male gender, and two key themes were generated from the review of self‐reported factors associated with this prescribing: relationships and care pathways.

Although the primary studies identified were reasonably well‐designed, they are limited by two main issues. First, the relatively small sample size of a large proportion of the studies, particularly regarding patient and carer perspectives (total patients *n* = 27, total informal carers *n* = 0). Second, even those quantitative studies with larger sample sizes must be interpreted cautiously due to the observational nature of the identified studies. Many of these studies also relied on data from electronic health records, which could have introduced confounding variables due to unreported patient factors. Despite identifying potential factors associated with prescribing in BPD, due to these limitations, this synthesis represents only a starting point in further exploring prescribing decisions in BPD.

Interestingly, patients and HCPs have reported similar beliefs surrounding the management of BPD. Both parties identified ‘relationships’ as integral to the prescribing process, though their beliefs around those relationships differed. What does emerge is the idea of a critical relationship between HCPs and their patients; the patient wants something and, to a lesser or greater degree, relies on the prescriber to obtain it.

This belief around the importance of, or need for, strong relationships has been reported in other studies about medication in patients with other serious mental illnesses (Green et al. 2008; Howe et al. 2023). Some of the relationships most valued by patients were those where the HCP showed care and treated clinical appointments ‘like friendships’ (Green et al. 2008), reflecting that often, patients seek more than medical treatment; they seek a sense of empathy and mutual trust (Maidment et al. 2011) and understanding from HCPs (Kerasidou et al. 2021).

In this review, multiple instances were found of patients referring to the importance of medication, treatment and validation and expecting these from interactions with HCPs. Alongside this, multiple patients expressed frustration with limited access to treatment and therefore sought medication as an available treatment, due to limited access to longer term psychotherapy. Thus, prescribing can be seen as an attempt to address an unmet treatment need. This act, even if not entirely aligned with best practices, may serve to validate patients' experiences and convey a sense of acknowledgment and care.

A previous study that examined the experiences of patients with medication in BPD found that medication was seen positively by patients as they were able to exert control over prescribing decisions. This was particularly noted in patients who transitioned to specialist services where they were given more autonomy around medication (Rogers and Acton 2012). The ability to exert influence may foster a sense of agency, which may improve outcomes in itself (Konstantinidou et al. 2023). Moreover, active involvement in decision‐making may serve as evidence of mutual trust, thereby strengthening the patient–HCP relationship. Therefore, given the importance of the prescriber–patient relationship, the observed levels of prescribing may reflect an attempt to validate patients' needs, thereby reinforcing the relationship.

However, it is worth noting that being prescribed medication is not validating for all patients. In some cases, patients felt that their concerns or other needs were rather dismissed through the act of prescribing (Rogers and Acton 2012; Tennant et al. 2023). It is important to note that the results detailing patients' perspectives here are based on only two qualitative studies, one of which focused predominantly on clozapine prescribing, therefore limiting any conclusions around patients' needs or wants and how these influence prescribing.

Studies in other cohorts of SMI patients have indicated HCP–patient relationships can be improved through reducing ‘mistrust’ (Howe et al. 2023). This concept is in line with a 2011 study employing focus groups on a number of mental health conditions in multiple mental health settings in England, which identified ‘trust’ as a key and critical component in medication management (Maidment et al. 2011) and therefore the care pathway as a whole. Based on our findings, it may be that one factor in reducing mistrust may be in the open engagement of patients in prescribing decisions. The treatment of BPD may present a unique challenge in patient engagement however, as many of the diagnostic criteria for BPD are indicative of difficulties in forming and maintaining well‐functioning relationships, and thus, the nature of the HCP‐patient relationship may differ in BPD.

Additionally, although not an identified theme, it is important to acknowledge that the diagnosis of BPD is one that can result in specific patterns of stigmatising behaviour by some HCPs (Baker and Beazley 2022), potentially adding further complications to a strained HCP‐patient relationship. This stigma will also need to be addressed when considering how a care pathway for BPD should be navigated, including prescribing decisions.

The idea that patients' expectations of being prescribed medication influence the decision to prescribe is neither a new phenomenon nor unique to BPD patients. A 2014 survey found that 55% of GPs felt under pressure to prescribe antibiotics. Furthermore, 44% of respondents disclosed that they had prescribed antibiotics to get a patient to leave the surgery, even if they did not believe them to be necessary (Cole 2014).

Patients' influence on prescribing decisions is also not isolated to primary care. A 2011 study of prescribing decisions in hospitals found that ‘pressure from patients, relatives, or carers was an uncomfortable influence on these hospital prescribers' prescribing decisions’. This theme was reported by all specialities and grades of medical prescribers (Lewis and Tully 2011).

When exploring the influence of the HCP–patient relationship in prescribing, it is also important to acknowledge the desires of prescribers. This review found recurrent expressions of feelings of ‘helplessness’ or ‘frustration’ on the part of HCPs. These feelings may lead to a dynamic where the use of medication aims to manage not only patients' needs but also the prescriber's emotions. In essence the desire to help or resolve distress leads to prescribing, sometimes referred to as ‘countertransference prescribing’ (Shapiro‐Thompson and Fineberg 2022). This risk of countertransference prescribing may be exacerbated by the well‐documented challenges in accessing long‐term psychotherapy, recommended for the management of BPD, arising from a number of issues including a shortage of trained providers, stigmatisation and financial or commissioning barriers (Shapiro‐Thompson and Fineberg 2022). HCPs are therefore left in a difficult position, where they are unable to follow guidance to offer psychotherapy and, feeling there are no alterative options, are left with prescribing.

The challenges around accessing treatment are entwined in the second emergent theme of the ‘care pathway’, along with the impacts of guidelines, referrals and specialist input on prescribing decisions raised by HCPs. Our findings identified several fundamental issues with the implementation of the guidelines. These include a lack of flexibility or discretion within the guidelines, limited access to long‐term psychological therapies, and the prevailing belief among HCPs and patients that the symptoms of BPD can be effectively managed through medication.

A 2018 review of 33 studies concluded, among other findings, that national guidance is fundamental to the prescribing process (Davari et al. 2018). As previously noted, the current preeminent guidelines advise against the use of medication in BPD, outside of comorbid conditions. Despite this clear position in the guidelines, repeated studies show high levels of prescribing in BPD (Bridler et al. 2015). A 2018 review suggests that guidelines that limit HCPs' discretion have ‘doubtful implementation’ (Davari et al. 2018). In the case of BPD guidelines, HCPs' discretion is almost entirely removed, except for the treatment of comorbid conditions. This may be exacerbated by a lack of long‐term psychotherapy options and potential concerns around the strain on the HCP–patient relationship if something is not offered (Shapiro‐Thompson and Fineberg 2022; Soler et al. 2022). This may go some way to explaining the divergence from guidance in prescribing in BPD.

It may be particularly difficult for HCPs to navigate the care pathway for BPD patients when, regardless of the evidence base upon which guidelines are produced, as previously discussed some patients believe that medication has or will help them (Patel and Konstantinidou 2020). As noted, the HCP–patient relationship appears, at times, to be complicated by feelings of ‘helplessness’ on the side of the HCP, who holds the perceived power. Therefore, in instances where medication is not prescribed, despite a patient's desire for it, an already complicated HCP–patient relationship may be further strained, creating a gap between patient expectations and HCP duties to provide evidence‐based care. This gap may be further influenced by the quality of the underlying evidence base. A 2022 Cochrane review evaluated the evidence supporting trials as largely of low or very low quality and concluded with low certainty that medication may result in no difference in any primary outcome (Stoffers‐Winterling et al. 2022). However, the reviewers also reported inconclusive effects on secondary outcomes. These secondary outcomes include, but are not limited to, chronic feelings of emptiness, affective instability and anger. If it is the case that the outcomes measured in trials are not the most important to HCPs, this could help explain the observed dichotomy.

In cases where a decision to prescribe is made as part of the BPD care pathway, it is clear that there is an absence of guidance for HCPs. Without this fundamental influence, HCPs are likely to defer back to personal experience, senior colleagues' advice and perhaps guidance for other mental health conditions.

In terms of personal experience and senior colleagues' advice, there are indications that HCPs prescribe for patients with BPD based on symptoms (Crawford et al. 2011; Pascual et al. 2010, 2021). As such, it would appear that, at least in part, some HCPs do not agree with the advice in the guidelines and feel that individual symptoms can be treated. This may be due to HCPs' previous positive experiences of improvement in BPD patients prescribed medication.

It can therefore be hypothesised that in some cases, given the importance of guidelines, HCPs may resort to guidelines meant for other mental health conditions when treating the symptoms of BPD patients. As the use of medications is especially common in BPD patients with comorbid conditions, HCPs may treat comorbid conditions or particular subthreshold symptoms of BPD itself. Treating for particular symptoms assumes a shared aetiology for every presentation of a symptom, which may not be true. For example, instances of low mood in patients with BPD are not explicitly indicative of depression and therefore should not be treated as such.

Furthermore, in cases where pharmacological treatments of BPD prove ineffective, which is not unlikely given the limited evidence for their efficacy (Stoffers‐Winterling et al. 2022), HCPs may adjust or escalate treatment according to the guidelines in place for the symptom or perceived comorbid condition. This hypothesis may explain the levels of prescribing in the treatment of patients with BPD, as patients can fail to respond to the initial treatment, potentially leading HCPs to explore augmentation and multiple medications to address patients' symptoms in line with guidelines for other mental health conditions.

### 4.1 Strengths and Limitations

This systematic review presents a comprehensive assessment of the current literature regarding the factors influencing prescribing in BPD patients. It identified 13 papers with diverse methodologies and populations, limiting the scope for statistical analysis. The included studies did, however, cover a range of practice settings, including primary, secondary and specialist care. The review did not include studies not published in English, and excluded pre‐1994 literature, which may have led to a loss of some available data. Despite some potential limitations, including the small number of primary studies and a limited review of grey literature, this review is the first—to our knowledge—to draw upon the available literature to explore factors influencing prescribing patterns in BPD. The key finding is the lack of data and limited research in this area.

### 4.2 Recommendations for Further Research

The review identified a lack of research into the factors influencing prescribing in patients with BPD. Only two studies examining patient perspectives, which included 26 individuals, were found. Replication studies of the existing literature, especially qualitative interviews with patients, would support the robustness of existing findings.

No studies exploring informal carers' views were identified, possibly due to the limited focus on patient perspectives in the existing literature. Further studies exploring the opinions of carers would also add value.

Beyond this, further research is needed to explore how the relationship between professionals and patients, care pathways and patient demographics can impact prescribing. In particular, further exploration and investigation of targeted interventions impacting the identified themes and any resulting changes in prescribing levels would be of particular interest and value.

Furthermore, research aimed at identifying the trial outcomes that are most valued by patients and HCPs could also be beneficial. Such studies can provide critical insights into shared priorities, which are important for understanding prescribing decisions and ensuring that future trials align more closely with the needs and expectations of both patients and HCPs.

By highlighting the gaps in current research, this review provides a starting point for future investigations and interventions.

## 5 Conclusion

Findings from this review identify demographics that are potential influences on the prescribing of medication in BPD patients, including comorbidity, age and presenting symptoms. One key conclusion is the limited research on factors affecting prescribing in this population.

Furthermore, the review indicates that the themes of the HCP–patient relationship, identified by both HCPs and patients, and the care pathway are also important influences on the prescribing process. Within both themes, some potential challenges have been suggested, particularly ‘helplessness’ on the part of HCPs, the influence of guidelines and patients' expectations.

Addressing and strengthening the HCP–patient relationship seems integral to improving prescribing. Although more research is required, these results indicate a promising starting point for future research and interventions.

There are indications that HCPs may be prescribing in BPD for symptoms they believe can be treated; to maintain therapeutic relationships; due to countertransference; and due to a lack of accessible long‐term psychotherapy. However, this is based on a small number of studies, only two of which explored patients' opinions and none examining the perspectives of informal carers or family members.

Future research should seek to explore this further to provide HCPs with insight into the factors at play in prescribing for BPD patients. Only through a better understanding of how people arrive at prescribing decisions can unnecessary prescribing be avoided or corrected.

## Author Contributions

JC conceived of the presented idea. JC, IM and MJ developed the theory and methodology. The searches, study selection and data extraction were conducted by JC and SJ. IM and MJ encouraged JC and SJ in the review and subsequent analysis and supervised the findings of this work. All authors discussed the results and contributed to the final manuscript.

## Ethics Statement

The submitted manuscript represents a Systematic Review and was exempt from the ethical approval process at the University of Bath, as it only involved secondary analysis of anonymised data.

## Conflicts of Interest

The authors declare no conflicts of interest.

## Data Availability

Data availability is not applicable to this article, as no new data were created or analysed in this study.

## Appendix A

**Table:**

Section and topic	Item #	Checklist item	Location where item is reported
**Title**			
Title	1	Identify the report as a systematic review.	Yes – TITLE
**Abstract**			
Abstract	2	See the PRISMA 2020 for Abstracts checklist.	TBC
**Introduction**			
Rationale	3	Describe the rationale for the review in the context of existing knowledge.	Page 7
Objectives	4	Provide an explicit statement of the objective(s) or question(s) the review addresses.	Page 8
**Methods**			
Eligibility criteria	5	Specify the inclusion and exclusion criteria for the review and how studies were grouped for the syntheses.	Page 9 Page 11
Information sources	6	Specify all databases, registers, websites, organisations, reference lists and other sources searched or consulted to identify studies. Specify the date when each source was last searched or consulted.	Yes
Search strategy	7	Present the full search strategies for all databases, registers and websites, including any filters and limits used.	Page 8
Selection process	8	Specify the methods used to decide whether a study met the inclusion criteria of the review, including how many reviewers screened each record and each report retrieved, whether they worked independently, and if applicable, details of automation tools used in the process.	Page 10 + Prisma Diagram
Data collection process	9	Specify the methods used to collect data from reports, including how many reviewers collected data from each report, whether they worked independently, any processes for obtaining or confirming data from study investigators, and if applicable, details of automation tools used in the process.	Page 10
Data items	10a	List and define all outcomes for which data were sought. Specify whether all results that were compatible with each outcome domain in each study were sought (e.g., for all measures, time points, analyses), and if not, the methods used to decide which results to collect.	Appendix
	10b	List and define all other variables for which data were sought (e.g., participant and intervention characteristics, funding sources). Describe any assumptions made about any missing or unclear information.	Appendix
Study risk of bias assessment	11	Specify the methods used to assess risk of bias in the included studies, including details of the tool(s) used, how many reviewers assessed each study and whether they worked independently, and if applicable, details of automation tools used in the process.	Page 10
Effect measures	12	Specify for each outcome the effect measure(s) (e.g., risk ratio, mean difference) used in the synthesis or presentation of results.	Table 2 and 3
Synthesis methods	13a	Describe the processes used to decide which studies were eligible for each synthesis (e.g., tabulating the study intervention characteristics and comparing against the planned groups for each synthesis (item #5)).	Page 11
	13b	Describe any methods required to prepare the data for presentation or synthesis, such as handling of missing summary statistics, or data conversions.	Page 11
	13c	Describe any methods used to tabulate or visually display results of individual studies and syntheses.	6
	13d	Describe any methods used to synthesise results and provide a rationale for the choice(s). If meta‐analysis was performed, describe the model(s), method(s) to identify the presence and extent of statistical heterogeneity, and software package(s) used.	Page 11
	13e	Describe any methods used to explore possible causes of heterogeneity among study results (e.g., subgroup analysis, meta‐regression).	NA
	13f	Describe any sensitivity analyses conducted to assess robustness of the synthesised results.	NA
Reporting bias assessment	14	Describe any methods used to assess risk of bias due to missing results in a synthesis (arising from reporting biases).	NA
Certainty assessment	15	Describe any methods used to assess certainty (or confidence) in the body of evidence for an outcome.	NA
**Results**			
Study selection	16a	Describe the results of the search and selection process, from the number of records identified in the search to the number of studies included in the review, ideally using a flow diagram.	Prisma Diagram
	16b	Cite studies that might appear to meet the inclusion criteria, but which were excluded, and explain why they were excluded.	NA
Study characteristics	17	Cite each included study and present its characteristics.	Table 1
Risk of bias in studies	18	Present assessments of risk of bias for each included study.	Appendix MMAT
Results of individual studies	19	For all outcomes, present, for each study: (a) summary statistics for each group (where appropriate) and (b) an effect estimate and its precision (e.g., confidence/credible interval), ideally using structured tables or plots.	Table 2 & 3
Results of syntheses	20a	For each synthesis, briefly summarise the characteristics and risk of bias among contributing studies.	Page 25
	20b	Present results of all statistical syntheses conducted. If meta‐analysis was done, present for each the summary estimate and its precision (e.g., confidence/credible interval) and measures of statistical heterogeneity. If comparing groups, describe the direction of the effect.	NA
	20c	Present results of all investigations of possible causes of heterogeneity among study results.	Page 8 & 13
	20d	Present results of all sensitivity analyses conducted to assess the robustness of the synthesised results.	NA
Reporting biases	21	Present assessments of risk of bias due to missing results (arising from reporting biases) for each synthesis assessed.	NA
Certainty of evidence	22	Present assessments of certainty (or confidence) in the body of evidence for each outcome assessed.	Table 3
**Discussion**			
Discussion	23a	Provide a general interpretation of the results in the context of other evidence.	Page 23
	23b	Discuss any limitations of the evidence included in the review.	Page 25
	23c	Discuss any limitations of the review processes used.	Page 25
	23d	Discuss implications of the results for practice, policy, and future research.	Page 25/26
**Other information**			
Registration and protocol	24a	Provide registration information for the review, including register name and registration number, or state that the review was not registered.	Page 21
	24b	Indicate where the review protocol can be accessed, or state that a protocol was not prepared.	Page 21
	24c	Describe and explain any amendments to information provided at registration or in the protocol.	NA
Support	25	Describe sources of financial or nonfinancial support for the review, and the role of the funders or sponsors in the review.	NA
Competing interests	26	Declare any competing interests of review authors.	NA
Availability of data, code and other materials	27	Report which of the following are publicly available and where they can be found:template data collection forms; data extracted from included studies; data used for all analyses; analytic code; any other materials used in the review.	Page 21 and Appendix

*Source:* Page et al. (2021)

## Appendix B

### B1 Search Terms

((‘borderline state’ OR ‘borderline personality disorder’ OR eupd) AND (‘factors’ OR ‘influences’ OR ‘perspectives’ OR ‘experiences’ OR ‘doctor‐patient relationship’)) AND (prescribing OR ‘practice of prescribing’)

Whenever possible, the following filters will be used:

- Date (publication year) 1994–2023. A filter on the date of publication from 1994 to present will be utilised due to significant changes in diagnosis criteria for BPD in 1994, with the implementation of DSM‐IV.
- Languages. A filter restricted to English only, as the authors are limited to the English language and unable to properly analyse non‐English work.

## Appendix D

### D.1

**Table:**

No.	Item	Guide and description	Page
1	Aim	State the research question the synthesis addresses.	5
2	Synthesis methodology	Identify the synthesis methodology or theoretical framework which underpins the synthesis, and describe the rationale for choice of methodology (e.g., *meta‐ethnography*, *thematic synthesis*, *critical interpretive synthesis*, *grounded theory synthesis*, *realist synthesis*, *meta‐aggregation*, *meta‐study*, *framework synthesis*).	6,7
3	Approach to searching	Indicate whether the search was pre‐planned (*comprehensive search strategies to seek all available studies*) or iterative (*to seek all available concepts until they theoretical saturation is achieved*).	5
4	Inclusion criteria	Specify the inclusion/exclusion criteria (e.g., *in terms of population*, *language*, *year limits*, *type of publication*, *study type*).	5
5	Data sources	Describe the information sources used (e.g., *electronic databases* (*MEDLINE*, *EMBASE*, *CINAHL*, *psycINFO*, *Econlit*), *grey literature databases* (*digital thesis*, *policy reports*), *relevant organisational websites*, *experts*, *information specialists*, *generic web searches* (*Google Scholar*) *hand searching*, *reference lists*) and when the searches conducted; provide the rationale for using the data sources.	5
6	Electronic Search strategy	Describe the literature search (e.g., *provide electronic search strategies with population terms*, *clinical or health topic terms*, *experiential or social phenomena related terms*, *filters for qualitative research and search limits*).	5 & Appendices
7	Study screening methods	Describe the process of study screening and sifting (e.g., *title*, *abstract and full text review*, *number of independent reviewers who screened studies*).	6
8	Study characteristics	Present the characteristics of the included studies (e.g., *year of publication*, *country*, *population*, *number of participants*, *data collection*, *methodology*, *analysis*, *research questions*).	Table 1
9	Study selection results	Identify the number of studies screened and provide reasons for study exclusion (e.g., *for comprehensive searching*, *provide numbers of studies screened and reasons for exclusion indicated in a figure/flowchart; for iterative searching describe reasons for study exclusion and inclusion based on modifications the research question and/or contribution to theory development*).	Prisma Diagram 1
10	Rationale for appraisal	Describe the rationale and approach used to appraise the included studies or selected findings (e.g., *assessment of conduct* [*validity and robustness*], *assessment of reporting* [*transparency*], *assessment of content and utility of the findings*).	6
11	Appraisal items	State the tools, frameworks and criteria used to appraise the studies or selected findings (e.g., *Existing tools:CASP*, *QARI*, *COREQ*, *Mays and Pope* [25]*; reviewer developed tools; describe the domains assessed:research team*, *study design*, *data analysis and interpretations*, *reporting*).	6
12	Appraisal process	Indicate whether the appraisal was conducted independently by more than one reviewer and if consensus was required.	6
13	Appraisal results	Present results of the quality assessment and indicate which articles, if any, were weighted/excluded based on the assessment and give the rationale.	Appendices
14	Data extraction	Indicate which sections of the primary studies were analysed and how were the data extracted from the primary studies? (e.g., *all text under the headings “results/conclusions” were extracted electronically and entered into a computer software*).	6 & appendices
15	Software	State the computer software used, if any.	6
16	Number of reviewers	Identify who was involved in coding and analysis.	7
17	Coding	Describe the process for coding of data (e.g., *line by line coding to search for concepts*).	6, 7
18	Study comparison	Describe how were comparisons made within and across studies (e.g., *subsequent studies were coded into preexisting concepts*, *and new concepts were created when deemed necessary*).	7
19	Derivation of themes	Explain whether the process of deriving the themes or constructs was inductive or deductive.	6, 7
20	Quotations	Provide quotations from the primary studies to illustrate themes/constructs, and identify whether the quotations were participant quotations of the author's interpretation.	11–13
21	Synthesis output	Present rich, compelling and useful results that go beyond a summary of the primary studies (e.g., *new interpretation*, *models of evidence*, *conceptual models*, *analytical*)	13–15

## Appendix E

**Table:**

Reference	1. Qualitative studies				
	1.1. Is the qualitative approach appropriate to answer the research question?	1.2. Are the qualitative data collection methods adequate to address the research question?	1.3. Are the findings adequately derived from the data?	1.4. Is the interpretation of results sufficiently substantiated by data?	1.5. Is there coherence between qualitative data sources, collection, analysis and interpretation?
Wlodarczyk et al. (2018)	Yes	Yes	Yes	Cannot tell	Yes
Martean and Evans (2014)	Yes	Yes	Yes	Cannot tell	Yes
Patel and Konstantinidou (2020)	Yes	Yes	Yes	Cannot tell	Yes
Dickens et al. (2016)	Yes	Yes	Yes	No	Yes

**Table:**

Reference	4. Quantitative Descriptive Studies				
	4.1. Is the sampling strategy relevant to address the research question?	4.2. Is the sample representative of the target population?	4.3. Are the measurements appropriate?	4.4. Is the risk of nonresponse bias low?	4.5. Is the statistical analysis appropriate to answer the research question?
Pascual et al. (2010)	Yes—for specialist service	Yes—for specialist service	Yes	NA	Yes
Tong et al. (2021)	Yes—for specialist service	Yes—for specialist service	Yes	NA	Yes
Pascual et al. (2021)	Yes	Yes	Yes	NA	Yes
Knappich et al. (2014)	Yes	Yes	Yes	No	Yes
Schulkens et al. (2021)	Yes	Yes	Yes	Yes	Yes
Javed et al. (2022)	Yes	Yes	Yes	Cannot tell	Yes
Pascual et al. (2007)	Yes	Yes	Yes	Yes	Yes
Crawford et al. (2011)	Yes	Yes	Yes	Yes	Yes
Mirhaj Mohammadabadi et al. (2022)	Yes	Yes	Yes	Cannot tell	Yes
